# Commentary: A Possible Mechanism of Zika Virus Associated Microcephaly: Imperative Role of Retinoic Acid Response Element (RARE) Consensus Sequence Repeats in the Viral Genome

**DOI:** 10.3389/fmicb.2018.00190

**Published:** 2018-02-20

**Authors:** Muneeb A. Faiq, Ashutosh Kumar, Himanshu N. Singh, Vikas Pareek, Pavan Kumar

**Affiliations:** ^1^Dr. Rajendra Prasad Centre for Ophthalmic Sciences, All India Institute of Medical Sciences, New Delhi, India; ^2^Laboratory for Molecular Reproduction and Genetics, Department of Anatomy, All India Institute of Medical Sciences, New Delhi, India; ^3^Medical Biotechnology Laboratory, Dr. B. R. Ambedkar Centre for Biomedical Research, University of Delhi, New Delhi, India; ^4^Etiologically Elusive Disorders Research Network, New Delhi, India; ^5^Department of Anatomy, All India Institute of Medical Sciences, New Delhi, India; ^6^Functional Genomics Unit, Institute of Genomics and Integrative Biology, New Delhi, India; ^7^Department of Biochemistry, All India Institute of Medical Sciences, New Delhi, India; ^8^Computational Neuroscience and Neuroimaging Division, National Brain Research Centre, Manesar, India

**Keywords:** endogenous retroviruses, retinoic acid signaling, retrovirus, reverse transcriptase, RNA virus, zika virus

“If at first the idea is not absurd, then there is no hope for it” - Albert Einstein

The present commentary emerges from the skepticism about our recent paper describing/proposing the mechanism of Zika-virus (a positive strand RNA virus) mediated microcephaly (Kumar et al., 2016). Our hypothesis mainly elaborates on the retinoic acid signaling problems precipitated by Zika infection. Owing to our intriguing bioinformatics results, we commenced with a coherent line of arguments that viral DNA (somehow synthesized from the viral RNA) may be integrating into the host DNA, and if the inserted DNA sequences match the regulatory regions of certain host genes then their expression signature may be altered. It could then be justified that the Retinoic Acid Response Element (RARE) consensus sequence repeats (if present) in the genomic sequences of ZIKV strains would be inserted into the regulatory regions of RARE dependent genes of the host DNA and, therefore, could influence their expression in a way that the developing fetus manifests with brain malformation like microcephaly. Another reason for implicating RARE elements in Zika induced microcephaly is because retinoic acid metabolism has an imperatively essential role in normal brain development, and the reported cases of Zika virus mediated microcephaly demonstrated striking resemblances to retinoic acid embryopathy (Kumar et al., 2016; Mawson, 2016). The most plausible explanation that could account for all the features of Zika microcephaly was through retinoic acid signaling. All this was the substance of our paper published last year (Kumar et al., 2016) which necessitates the possibility of integration of a positive strand RNA virus genome into the host genome (POSTRAITE: POsitive STrand RnA Integration to hosT gEnome) as a starting premise. We will, therefore, be discussing this possibility as a central idea in the present commentary. Also, it has come to our notice that some investigators may have concerns regarding the premise of POSTRAITE because positive strand RNA viruses neither have a reverse transcriptase nor an integrase. To lay this doubt to rest, here we provide a cogent rationale about how our premise is coherent and how there is a possibility for positive strand RNA viruses to integrate into the host genome.

Given the utmost importance of understanding the mechanism of certain important viral diseases (including Zika virus mediated microcephaly), the present anecdote aims at analyzing POSTRAITE as a possibility. Such a possibility may be considered a remote prospect by majority of virologists because of the prevailing perspective that positive strand RNA viruses do not need to synthesize DNA to complete their life cycle neither do they have any reverse transcriptase to generate a complementary DNA. Since there seems to be no chance of reverse transcription (in the light of the absence of reverse transcriptase in the viral genome), it becomes easy to infer that a DNA stage (i) does not exist and (ii) is not functionally important. We argue that these inferences are not true. The current paradigm is that DNA transcribed from the RNA of these viruses does not exist in the host genome; and it requires a robust evidence of POSTRAITE to refute this generally held belief. In this paper we will demonstrate that such affirmation already exists.

Scientific literature is filled with papers that don't even consider the possibility of RNA to DNA synthesis in these viruses and their host genome integration. But we propose that there is a genuine likelihood despite the absence of reverse transcriptase and integrase in their genomes. In order to give a scientific basis to this apparently radical proposition, we need to establish atleast one obligatory and one supportive postulate viz. (i) experimental evidence of a positive strand RNA viral genome (devoid of reverse transcriptase and integrase) integration into host genome (obligatory) and (ii) speculation of possible function/s such an integration may serve the virus (supportive). If, there is evidence of the virus antigens being found in the nuclei of the host cells, our proposition may imbibe further strength. The mechanism of host genome integration, in principle, can be true with many other diseases where the viral genome has sequence based similarities to certain regions of the host genome. This conceptual model is coherent and may pave way to deciphering mechanisms of many complex viral diseases (in addition to Zika virus mediated brain defects). But for such a mechanism to be true, POSTRAITE needs to be a biological verity. Establishing the likelihood of such a biological reality is the basic aim of the present paper and can be achieved by finding satisfactory answers for the above mentioned obligatory and supporting postulates.

Positive strand RNA viruses are indeed RNA-to-Protein dominant viruses, and (as of now) have not been reported to reverse transcribe into cDNA. As an example of Zika virus, an RNA polymerase on the NS5 subunit of the Zika virus polyprotein has been indicated for viral genome replication (RNA-dependent-RNA polymerase activity), without the involvement of reverse transcriptase (Godoy et al., 2017). Therefore, it becomes difficult to imagine a DNA stretch of the viral genome to integrate into the host genome. But this deduction is not absolute. Endogenous retroviruses—the viruses embedded throughout mammalian genomes accounting for almost 8% of the human genome (Tyagi et al., 2017)—might provide a molecular pathway to these RNA viruses to integrate into host genomes. This possibility has been experimentally proved by Geuking et al. (2009) and (in *Aedes* mosquito genomes) by Suzuki et al. (2017). Their findings are interesting, run counter to existing paradigm and support our proposition of genome to genome integration even in positive strand RNA viruses. Additionally, there is evidence that these archived endogenous retroviruses encode active reverse transcriptases (Berkhout et al., 1999). This indicates that these RNA viruses, as per one speculation, may induce the expression of endogenous retroviral reverse transcriptases leading to a sort of “Genome Resurrection.” This, in turn, may surmise the presence of (what can be called) “Endogenous Retrovirus Resurrection Elements” in the positive strand RNA viruses. But we emphasize that these elements are, as of now, suppositious.

In an interesting study, Klenerman et al. (1997) reported a small fraction (around 1 in 10,000–100,000 splenocytes) of mouse cells to possess DNA that was complimentary to mouse RNA viruses. These cells were possessed by what Weiss and Kellam (1997) referred to as “Illicit Viral DNA.” Klenerman group observed that the RNA virus LCMV had, by some unknown mechanism, integrated in the mouse genome. Though surprising, this strongly indicates that there is a possibility of POSTRAITE and establishes a scientific basis for the phenomenon. Furthermore, Hangartner from the same group established two cell lines which contained DNA copies integrated from the LCMV. He speculated that the LCMV might be interacting through the intervention of mouse endogenous retroviruses (Geuking et al., 2009). This is a possibility for Zika virus also and strongly bolsters the premise on which our hypothesis is based. Also, studies by Geuking et al. (2009) and Berkhout et al. (1999) provide a scientific basis to our proposed mechanism of ZIKV mediated microcephaly.

RNAs are relatively unstable molecules; for that reason the genomes of RNA viruses are susceptible to various host cell mechanisms aimed at exonucleolytic mRNA decay/destruction (Moon et al., 2012). Eukaryotic cells have rigorous machinery for rapid degradation of unwanted RNA transcripts arising from many processes (Clark et al., 2012; Moon et al., 2012). The transcripts that arise from these RNA viruses belong to the “to be destroyed” category because viral transcripts generally have different ribonucleoprotein organization and lack a cap region and a poly(A) tail (Moon et al., 2012). Additionally, the RNA dependent RNA polymerase of these viruses has low fidelity which serves an important function of increasing the error rate in order to evade the immune system (Diamond, 2003). As a result, the RNA genome does not exist as a single transcript but as a multitude of related transcripts. Though this may be a defense mechanism but also comes with a tradeoff of drifting away from the original transcript and consequent species extinction (Lauring et al., 2013). A possible mechanism to avoid species extinction would be to integrate the virus genome into host genome in DNA form, although we acknowledge this scenario is highly speculative. This aim will be relatively easy with those cells that have actively replicating genome (i.e., the dividing cells). That may be the reason why undifferentiated neurons (at early neurogenesis stages in fetal brains) are extremely susceptible to ZIKV infections while well differentiated adult neurons, are relatively resistant (Hughes et al., 2016). Genome archiving may, nevertheless, be relevant and relatively easier aim for retroviruses also, thereby opening novel prospects to our understanding of diseases like AIDS.

Though the complete lifecycle of positive strand RNA viruses is thought to be exclusively cytoplasmic, their presence in the host cell nuclei have, nevertheless, been documented (Buckley and Gould, 1988; Lopez-Denman and Mackenzie, 2017). Buckley and coworkers observed fluorescent staining by viral envelope glycoprotein (MAb-541) in the nuclei (but not the cytoplasm) of cells infected with Zika virus. They also observed NS1 glycoprotein (MAb-109) staining exclusively in the nucleoli of cells infected with Langat virus (though nuclear localization of genome is yet to be confirmed). Using Double-labeling strategies, they further confirmed that the nuclear staining was restricted to virus-infected cells only. This brings into question the belief of cytoplasm-centric lifecycle of positive strand RNA viruses and reinforces the notion of genome integration.

Though the supposition of positive strand RNA virus genome integration to host genome is currently considered improbable but this phenomenon is, nevertheless, a reality and has already been reported. This concept has a lot of information to offer from safety concerns of RNA based gene therapies to viral etiology of previously thought non-infectious diseases. The etiobiology of neurodegenerative disease may further be understood under this umbrella (Li et al., 2015). And relapse of a disease after complete virus neutralization may also be explained. While we agree that this commentary has a strong speculative element but our proposition is supported by experimental evidence, peer reviewed literature and comprehensively rational line of arguments. Coming decades are likely to provide convincing and useful answers.

## Author contributions

MF and AK: conceived the idea, wrote the manuscript, and revised the manuscript; HS, VP, and PK: wrote and revised the manuscript.

### Conflict of interest statement

The authors declare that the research was conducted in the absence of any commercial or financial relationships that could be construed as a potential conflict of interest.
